# Application of Multiple-Optimization Filtering Algorithm in Remote Sensing Image Denoising

**DOI:** 10.3390/s23187813

**Published:** 2023-09-12

**Authors:** Xuelin Zhang, Yuan Li, Xiang Feng, Jian Hua, Dong Yue, Jianxiong Wang

**Affiliations:** University Research Center of Agricultural Remote Sensing and Precision Agriculture Engineering in Yunnan Provincial, School of Water Conservancy, Yunnan Agricultural University, Kunming 650201, China; zxl1947253858@163.com (X.Z.); ly1006527@163.com (Y.L.); fengxiang_mys@163.com (X.F.); 15614106896@163.com (J.H.); 18391917875@163.com (D.Y.)

**Keywords:** remote sensing imagery, Gaussian noise, edge detection operator, differential evolution algorithm, bilateral filtering

## Abstract

Denoising remote sensing images is crucial in the application and research of remote sensing imagery. Noise in remote sensing images originates from sensor characteristics, signal transmission, and environmental conditions, among which Gaussian noise is the most common type. In this paper, we proposed a multiple-optimization bilateral filtering (MOBF) algorithm based on edge detection and differential evolution (DE) methods. The proposed algorithm optimizes the spatial domain filtering kernel and the spatial domain Gaussian kernel by using the standard deviation and width of the edge response. By employing the DE algorithm, the individuals in the population based on the standard deviation of the gray value domain are subjected to iterative mutation, crossover, and selection operations to refine the latent solution vectors and determine the optimal color space for optimizing the standard deviation of the pixel range domain kernel. As a result, the MOBF algorithm, which does not require any parameter input, is realized. To verify the feasibility and effectiveness of the proposed algorithm, denoising experiments were conducted on remote sensing images by using evaluation metrics such as the mean squared error, peak signal-to-noise ratio, and structural similarity index. The experimental results revealed that the MOBF algorithm outperforms traditional algorithms for all three evaluation metrics.

## 1. Introduction

Remote sensing imagery is widely used in various fields, such as geological exploration, urban planning, and environmental monitoring. However, various noise sources, such as circuit noise and dark current noise [1], can introduce Gaussian noise into remote sensing imagery. In addition, noise interference during digital image transmissions and storage can result in salt-and-pepper noise. These noise sources degrade image quality, affecting subsequent analyses and applications [2]. Therefore, denoising is crucial for processing remote sensing images.

Denoising methods for remote sensing images typically employ linear or nonlinear filtering techniques, such as median filtering [3], Gaussian filtering [4], or a wavelet transform [5]. However, these methods have certain limitations. For instance, median filtering can result in image distortion [6], and a wavelet transform involves complex decomposition and reconstruction operations [7]. The bilateral filter, proposed by Tomasi and Mandeville [8] in the 1980s, has undergone improvements and developments and has emerged as a suitable method for image denoising and edge preservation. Chen et al. [9] proposed a denoising approach by combining a wavelet transform and bilateral filtering. They utilized multilevel thresholding based on a wavelet analysis to handle mixed noise and effectively eliminate noise at different frequencies in the image. Deng et al. [10] integrated adaptive parameter bilateral filtering with a wavelet transform to improve image retrieval. They used the Corel-5K image database for validation and achieved a high applicability. Lin [11] proposed the concept of gradient similarity to distinguish between edge blocks and non-edge blocks in image processing and improved bilateral filtering by employing gradient similarity instead of grayscale similarity. Zhang et al. [12] developed a novel two-step denoising method for handling mixed noise. They employed the DnCNN denoising model to reduce Gaussian noise and used an adaptive median filtering technique to reduce salt-and-pepper noise. Liu et al. [13] proposed an adaptive median filter with edge preservation wherein the edge is first extracted using an edge extractor, and then the median-filtered image is fused with the extracted edge to realize effective filtering and edge enhancement.

Bilateral filtering represents an extended form of the Gaussian filtering methodology. This technique incorporates two key factors during image processing: the spatial coordinates of pixels and the disparities in pixel intensities. This amalgamation enables the enhanced preservation of edge data [14]. In contrast, conventional Gaussian filtering exclusively addresses the spatial arrangement of pixels by employing a Gaussian kernel to execute the weighted averaging of neighboring pixels, thereby facilitating a resultant blurring outcome. Notably, the inherent limitation of conventional Gaussian filtering resides in its omission of intensity discrepancies among pixels. The advent of bilateral filtering introduces an additional layer of weighting within the intensity domain, superimposed upon the principles of Gaussian filtering. This approach not only considers the spatial separation between pixels, but also takes into careful consideration the disparities in pixel intensities. As a result, bilateral filtering achieves a dual objective: the retention of edge information alongside the realization of smoothing effects.

In this paper, we proposed a remote sensing image denoising method based on multi-objective optimized bilateral filtering. The proposed method employs several techniques to enhance the bilateral filtering parameters. It combines edge detection operators and the differential evolution (DE) algorithm with traditional bilateral filtering. The adjusted parameters include the convolution kernel size, the spatial standard deviation of pixel positions, and the color space standard deviation of pixel values. These modifications greatly improve the noise reduction capabilities of the proposed method. Furthermore, the implementation process and experimental results of the proposed method are discussed in this paper. The results demonstrated a substantial enhancement in denoising performance while preserving the edges, thus indicating that the proposed method is suitable for subsequent processing and applications of remote sensing images.

## 2. Principles and Methods of Filtering Algorithms

### 2.1. Bilateral Filtering

Bilateral filtering is a powerful nonlinear filtering method that eliminates noise and preserves edge details in an image. It is based on a weighted average of the image’s spatial domain and grayscale value domain. The weights are determined based on the product of a spatial domain Gaussian kernel and a grayscale value domain Gaussian kernel [15,16]. By considering the spatial distance between pixels and the disparity in grayscale values, bilateral filtering assigns appropriate weights, resulting in the improved preservation of edges and fine details. The formula for bilateral filtering is as follows:

(1) $$G_{d}=e^{-{\frac{d(p(x,v),q(x,y))}{2{\sigma_{d}}^{2}}}^{2}},$$

(2) $$G_{s}=e^{-{\frac{∥I_{i}-I_{j}∥}{2{\sigma_{s}}^{2}}}^{2}},$$

(3) $$W_{p,q}=\sum_{p,q\in S}G_{d}G_{s}$$

where $G_{d}$ represents the spatial domain Gaussian kernel, $G_{r}$ denotes the grayscale value domain Gaussian kernel, $W_{p,q}$ is the weight, $d\left(p\left(x,v\right),q\left(x,y\right)\right)$ is the spatial distance between pixels (the Euclidean distance between pixels in an image), ${∥I}_{i}-I_{j}∥$ denotes the color difference between pixels $I_{i}$ and $I_{j}$, $\sigma_{d}$ is the spatial standard deviation, and $\sigma_{s}$ is the color space standard deviation.

### 2.2. Canny Operator

The Canny edge detection algorithm is a multistage edge detection algorithm proposed by John F. Canny in 1986. This algorithm processes single-channel grayscale images by detecting the first and second derivatives of pixel intensities and determines whether a pixel is located on an edge [17,18].

The algorithm involves the following steps. Gaussian filtering is applied to the input grayscale image to effectively remove noise:

(4) $$G(x,y)=\frac{1}{2\pi \sigma^{2}}e^{-\frac{x^{2}+y^{2}}{2\sigma^{2}}},$$

where (*x*, *y*) denote the coordinates of the filter and *σ* denotes the standard deviation of the Gaussian function.

Next, the gradient magnitude and direction are computed for each pixel in the image:

(5) $$\theta =\arctan\frac{G_{x}(x,y)}{G_{y}(x,y)},$$

(6) $$I(x,y)=\sqrt{{\left(G_{x}(x,y)\right)}^{2}+{\left(G_{y}(x,y)\right)}^{2}}$$

Subsequently, non-maximum suppression is applied to the gradient magnitude to reduce the width of the detected edges.

The gradient magnitude is categorized into three groups by using a dual threshold algorithm: strong edges, weak edges, and non-edge pixels. Strong edges have gradient magnitudes surpassing the high threshold, weak edges have gradient magnitudes falling between the low and high thresholds, and non-edge pixels have gradient magnitudes below the low threshold. Furthermore, edge connectivity processing is employed for the weak edges to detect weak edge pixels adjacent to strong edges and upgrade their classification to strong edges. The integration process establishes connections among the weak edges, resulting in a comprehensive and refined edge detection outcome.

To determine the upper and lower thresholds for the Canny algorithm, an automated technique known as the Otsu method is applied. This method serves to establish the binarization threshold of the image, a crucial step in the process of image binarization segmentation. The Otsu method autonomously identifies a suitable threshold for binarization, thereby enabling the clear extraction of target object contours [19,20]. The Otsu algorithm’s core steps encompass the following:

The image’s histogram is computed, tabulating the occurrences of grayscale levels for each pixel.

The within-class variance is calculated by categorizing pixels into two groups, namely the background and the foreground, based on each possible threshold value “*t*”. The between-class variance $\sigma_{k}^{2}(t)$ is calculated for each “*t*” according to the formula:

(7) $$\sigma_{k}^{2}(t)=\omega_{0}(t)\omega_{1}(t){\left(\mu_{0}(t)-\mu_{1}(t)\right)}^{2}$$

Here, “*k*” signifies the two categories 0 and 1; “*ω*” denotes the proportion of pixels with grayscale values less than or equal to “*t*” in the “*k*”-th class; and μ(t) represents the average grayscale value of pixels with grayscale values less than or equal to “*t*” in the “*k*”-th class.

The global inter-class variance $\sigma_{B}^{2}(t)$ is calculated.

(8) $$\sigma_{B}^{2}(t)=\sum_{i=0}^{L-1}p(i){\left[\mu_{T}-\mu_{i}(t)\right]}^{2}$$

The optimal threshold is identified by selecting from all feasible thresholds the one that maximizes the inter-class variance g(t). By undergoing this computation, the Otsu algorithm automatically pinpoints the most appropriate binarization threshold, thereby enhancing the distinction between the foreground and the background and effectively facilitating object extraction from the image. In this study, the high and low thresholds for the Canny algorithm utilize binarization thresholds determined by the Otsu algorithm, scaled by 0.5 and 1.5 times.

### 2.3. Differential Evolution Algorithm

The DE algorithm is an optimization algorithm primarily used to solve multidimensional continuous optimization problems. It was proposed by Storn and Price in 1997 and has since become a widely used global optimization algorithm [21,22,23]. The fundamental concept of the DE algorithm is to iteratively improve a population of individuals by manipulating their differences. Through this iterative process, a new population is created, and the individuals within the population are progressively optimized. The DE algorithm mainly comprises the following steps:

First, an initial population, where each individual represents a potential solution, is randomly generated:

(9) $$x_{j,i,0}=x_{min}^{j}+{rand}_{j}(0,1)*\left(x_{max}^{j}-x_{min}^{j}\right),$$

where i= 1, 2, 3, …, *NP* (population size); j= 1, 2, 3, …, *D* (dimensionality); and ${rand}_{j}(0,1)$ denotes a uniformly distributed random number in the interval [0, 1].

The mutation operation randomly selects three distinct individuals and updates the current individual’s position by using the difference vectors between them, resulting in a new candidate solution:

(10) $$v_{i,g}=x_{r1,g}+F*\left(x_{r2,g}-x_{r3,g}\right),$$

where $r_{1},r_{2}$, and $r_{3}$ are distinct integers selected from the interval [1, *NP*].

During the crossover operation, the mutation vector is combined with the original individual to create a new individual. The target vector x is crossed with the mutation vector v to create the trial vector u:

(11) $$u=\left\{\begin{array}{l} v_{ij,g},\;\mathrm{if}\;\mathrm{rand}(0,1)\leq CR\;\mathrm{or}\;j=j_{\mathrm{rand}\;}\\ x_{ij,g},\;\mathrm{otherwise}\; \end{array}\right.,$$

where $j_{rand}$ is a random integer selected from the range [1, D] to ensure that $v_{ij,g}$ carries information. The crossover probability CR has a value between 0 and 1.

The selection operation involves comparing the trial individuals with the original individuals and selecting the superior individuals as members of the next-generation population:

(12) $$x_{i,g+1}=\left\{\begin{array}{l} u,\;\mathrm{if}\;f\left(u_{i,g}\right)>f\left(x_{i,g}\right)\\ x_{i,g},\;\mathrm{otherwise}\; \end{array}\right.,$$

where $f\left(x\right)$ represents the objective function.

The termination criteria for the DE algorithm are met when either the maximum predetermined number of generations is reached or when the objective function has converged to a considerable extent. At this stage, the algorithm concludes and outputs the optimal solution.

## 3. Multiscale-Optimized Bilateral Filtering

The parameters of the bilateral filter, such as the spatial domain filter kernel and the standard deviation of the spatial domain Gaussian kernel, are adjusted using the Canny operator. First, the Otsu thresholding technique is used to adjust the high and low thresholds (${T\;}_{h}$ and ${T\;}_{L}$, respectively) of the Canny edge detection operator:

(13) $${T\;}_{h}=1.5\times argmax\left\{\sigma_{B}^{2}(t)\right\},$$

(14) $${T\;}_{L}=0.5\times argmax\left\{\sigma_{B}^{2}(t)\right\}.$$

Strong edge pixels of an edge response are defined as I(x,y)>I(x,y), weak edge pixels of an edge response are defined as ${T\;}_{L}<I(x,y)\leq {T\;}_{h}$, and non-edge pixels are defined as $I(x,y)\leq {T\;}_{L}$.

The edge width is computed using non-maximum suppression:

(15) $$W\left(x,y\right)=\frac{I_{x}(x,y)}{I(x,y)}.$$

The standard deviation of the edge response image is computed as follows:

(16) $$\sigma =\sqrt{\frac{\sum_{i=1}^{n}{\left(x_{i}-\mu \right)}^{2}}{n}},$$

where *µ* denotes the population mean and *x_i_* represents the *i*-th sample data point.

The spatial domain Gaussian kernel $G_{d}$ is optimized:

(17) $$G_{d}=e^{-{\frac{d(p(x,v),q(x,y))}{2{\sigma_{d}}^{2}}}^{2}}=e^{-\frac{{∥p_{i}-p_{j}∥}^{2}2n}{\sum_{i=1}^{n}{\left(x_{i}-\mu \right)}^{2}}},$$

where $p_{i}$ and $p_{j}$ are the coordinates of pixel points i and j, respectively, and $\sigma_{space}$ is replaced by $\frac{\sigma }{2}$ (the standard deviation of the edge response image).

The spatial domain filter kernel *D* is optimized:

(18) $$D=\frac{2W\left(x,y\right)}{\sigma }=\frac{{2I}_{x}(x,y)}{I(x,y)\sqrt{\frac{\sum_{i=1}^{n}{\left(x_{i}-\mu \right)}^{2}}{n}}},$$

where $W\left(x,y\right)$ is the edge width and *σ* is the standard deviation of the edge response image.

The pixel range domain kernel $G_{s}$ is optimized by using the DE algorithm:

(19) $$G_{s}=e^{-{\frac{∥I_{i}-I_{j}∥}{2{\sigma_{color}}^{2}}}^{2}},$$

where ${∥I}_{i}-I_{j}∥$ denotes the color difference (calculated using the Euclidean distance) between pixels $I_{i}$ and $I_{j}$ and $\sigma_{color}$ denotes the standard deviation of the color space of the pixel values:

(20) $$\sigma_{color}=\sqrt{\frac{\sum_{i=1}^{n}{\left(x_{i}-\mu \right)}^{2}}{n}}.$$

A population of 20 individuals is randomly generated within the range of [*σ*, 2*σ*] and their parameter vectors are initialized. The crossover probability, mutation probability, and number of iterations are set as 0.5, 0.1, and 400, respectively. The fitness function peak signal-to-noise ratio (PSNR) is defined to assess the fitness of each individual by determining the PSNR value of the filtered image corresponding to each individual:

(21) $$x_{i,g+1}=\left\{\begin{array}{l} u,\;\mathrm{if}\;f\left(u_{i,g}\right)>f\left(x_{i,g}\right)\\ x_{i,g},\;\mathrm{otherwise}\; \end{array}\right.,$$

where f(.) represents the PSNR function.

Individuals with a better fitness are selected from the population, and genetic operators such as crossover and mutation operations are applied to them to generate new individuals. The population is then updated by replacing the individuals with the newly generated ones. This is constantly iterated, ultimately yielding the optimal parameters and their respective PSNR values.

**Figure 1:**
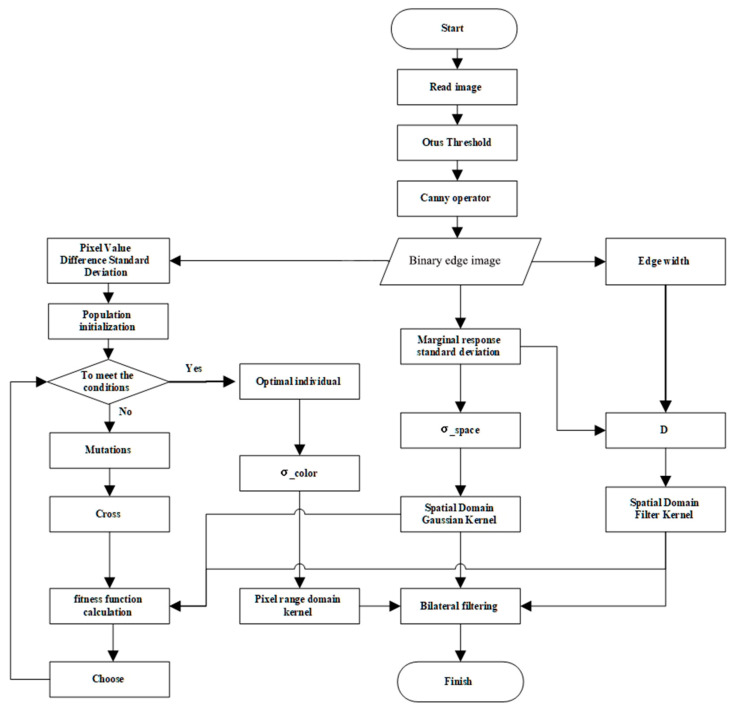
Flowchart of the multi-optimization bilateral filtering algorithm.

The flowchart of the multiscale-optimized bilateral filtering algorithm is illustrated in Figure 1.

Multiscale-optimized bilateral filtering offers several advantages. It adjusts the spatial domain Gaussian kernel and spatial domain filter kernel (D is the convolution kernel size) of the bilateral filtering based on the standard deviation and the edge response width of the Canny operator; moreover, it employs the DE algorithm to optimize the pixel range domain kernel. The advantages are listed as follows:

1. Edge preservation and smoothness control: The standard deviation of the Canny operator indicates the intensity variation in the edges of the image. By adjusting the size of the spatial domain Gaussian kernel based on the standard deviation of the Canny operator, the smoothness of the filter can be controlled. When the standard deviation of the Canny operator is larger, increasing the size of the spatial domain Gaussian kernel enhances the smoothing effect. In contrast, when the standard deviation of the Canny operator is smaller, reducing the size of the spatial domain Gaussian kernel helps preserve more details during the filtering process.
2. Noise suppression: The convolution kernel size (D) of bilateral filtering can be used to control the degree of noise suppression. A larger convolution kernel can effectively average the neighboring pixel values and reduce the effect of noise. By dynamically adjusting the convolution kernel size based on the standard deviation and edge response width of the Canny operator, an optimal kernel size that aligns with the intensity of the noise can be adaptively selected, enabling superior noise suppression.
3. High adaptability: The DE algorithm, which incorporates differential and mutation operations, enables a global search and the automatic selection of the optimal standard deviation for the pixel value color space based on the characteristics of the image. By considering the characteristics of the image, the DE algorithm selects the optimal standard deviation for the pixel value color space to adjust the pixel range domain kernel. This is achieved by combining it with the spatial domain Gaussian kernel and kernel size, enabling adaptive filtering.

## 4. Evaluation Criteria

### 4.1. Mean Squared Error

The mean squared error (*MSE*) is a commonly used metric for evaluating the denoising performance of image-processing algorithms. It is the average of the squared differences between the image pixel values and their corresponding true values. A smaller *MSE* indicates a more effective denoising result [24]:

(22) $$MSE=\frac{1}{M\times N}\sum_{i=1}^{M}\sum_{j=1}^{N}{\left[I\left(i,j\right)-J\left(i,j\right)\right]}^{2},$$

where *I* and *J* represent images of the same size with a width *W* and a height *H*, and $I\left(i,j\right)$ and $J\left(i,j\right)$ denote their pixel values, respectively.

The *PSNR* is a metric used for evaluating image quality [25]. It quantifies the ratio between the signal and the noise by using the maximum possible signal value as a reference. The *PSNR* is commonly used to compare the disparity in quality between an image and its post-processed version, such as after compression or denoising. The calculation formula for the *PSNR* is as follows:

(23) $$PSNR=10\times \log\left(\frac{255\times 255}{MSE}\right).$$

### 4.2. Structural Similarity Index

The structural similarity index (*SSIM*) is a standardized metric used for measuring the degree of similarity between two images. Unlike conventional metrics such as the *MSE* and *PSNR*, the *SSIM* better corresponds to the human visual perception of image quality [26]. It considers three aspects: luminance, contrast, and structure. The *SSIM* compares the mean, variance, and covariance of the pixel values in the two images, and these metrics are then weighted and aggregated to obtain the final similarity score. *SSIM* values range from −1 to 1, with a higher value indicating a stronger similarity between two images. An *SSIM* value of 1 means that the two images are perfectly identical.

(24) $$SSIM(x,y)=\frac{\left(2\mu_{x}\mu_{y}+c_{1}\right)\left(2\sigma_{xy}+c_{2}\right)}{\left(\mu_{x}^{2}+\mu_{y}^{2}+c_{1}\right)\left(\sigma_{x}^{2}+\sigma_{y}^{2}+c_{2}\right)},$$

where $\mu_{x}$ and $\mu_{y}$ denote the mean values of images x and y, respectively; $\sigma_{x}$ and $\sigma_{y}$ are the standard deviations of the pixel values in images *x* and *y*, respectively; $\sigma_{xy}$ denotes the covariance of the pixel values; and $c_{1}$ and $c_{2}$ are two constants introduced to avoid potential errors resulting from excessively small denominators that may lead to division by zero.

## 5. Experiment and Analysis

In this study, we conducted a remote sensing image denoising simulation experiment by using DF-1 satellite images and drone orthophotos with dimensions of 600 × 600 and 472 × 502 pixels, respectively. Gaussian noise was added to the images at four different concentration levels: 1%, 2%, 3%, and 4%. Subsequently, denoising experiments were performed, and the results were analyzed both qualitatively and quantitatively. A comparison of the denoising effectiveness of the multiple-optimization bilateral filtering (MOBF) algorithm and several other denoising techniques, namely anisotropic filtering (AF), adaptive median filtering (AMF), original bilateral filtering (BF), wavelet denoising (WD), and non-local means (NLM), was performed. The denoised images were evaluated using objective metrics such as the PSNR, the MSE, and the SSIM. Furthermore, an analysis of the noise power spectral density was conducted to determine the level of information preservation across different frequency domains, thus providing valuable insights into the effectiveness of the denoising techniques.

**Figure 2:**
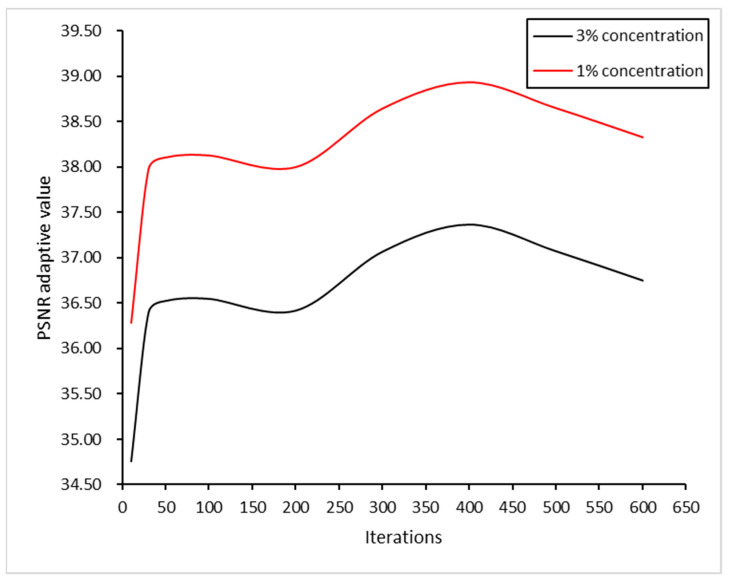
DE algorithm evolution parameters.

In order to find the optimal parameters of the DE algorithm, this paper conducts experiments under the conditions of a Gaussian noise concentration of 1% or 3%, and changes the iterative parameters of the DE algorithm to find the optimal parameters. The parameter of the iteration with the largest noise ratio (PSNR) is obtained through experiments. The optimal number of iterations is 400. Therefore, the number of iterations of the DE algorithm in the remote sensing simulation experiment in this paper is set to 400. The figure below shows the process of parameter optimization. The experimental results are shown in Figure 2.

### 5.1. GF-1 Image Simulation Experiment

**Figure 3:**
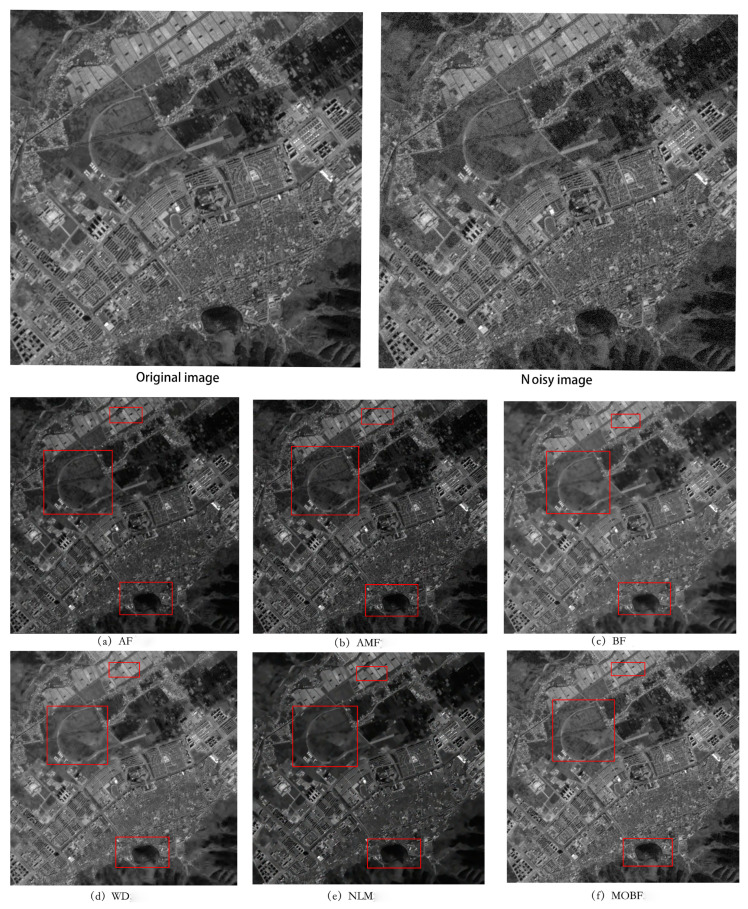
Comparison of GF-1 image denoising.

To validate the effectiveness of the proposed denoising algorithm, an experiment was conducted using GF-1 imagery for a remote sensing image simulation. A higher PSNR value indicated superior denoising results, reflecting a closer resemblance between the processed and original images. A smaller MSE value indicated a higher level of similarity in pixel values between the processed and original images, indicating minimal distortion. A higher SSIM value indicated an improved performance in terms of human perception, encompassing factors such as the brightness, contrast, and structural fidelity. The experimental results are presented in Table 1, and a denoising comparison of GF-1 images with a noise concentration of 4% is shown in Figure 3.

**Figure 4:**
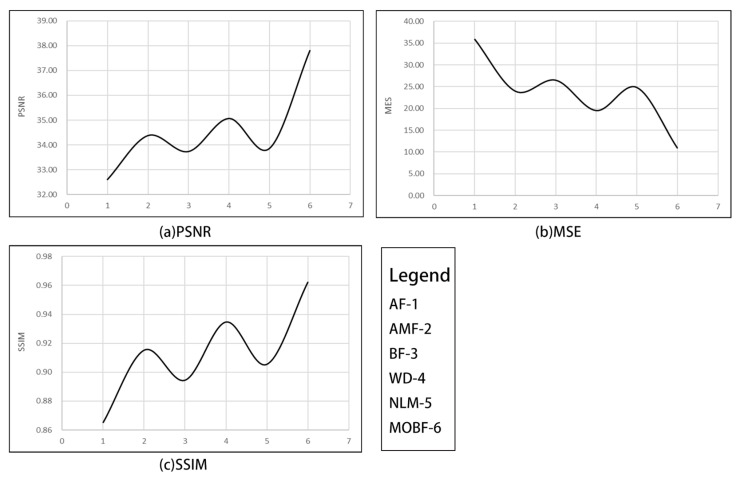
The average value of the evaluation indicators of each algorithm-*GF1*.

As can be seen from Figure 3, the MOBF algorithm outperformed other methods in terms of the denoising performance on GF-1 images. Notably, as can be seen in Figure 3f, the preserved texture details within the rectangular box region were visually more prominent than those obtained using other algorithms. Furthermore, as can be observed from the data presented in Table 1, the MOBF algorithm achieved the highest PSNR and SSIM values and the lowest MSE value across images with different noise densities. The algorithm performance is shown in Figure 4.

### 5.2. Unmanned Aerial Vehicle Image Simulation Experiment

**Figure 5:**
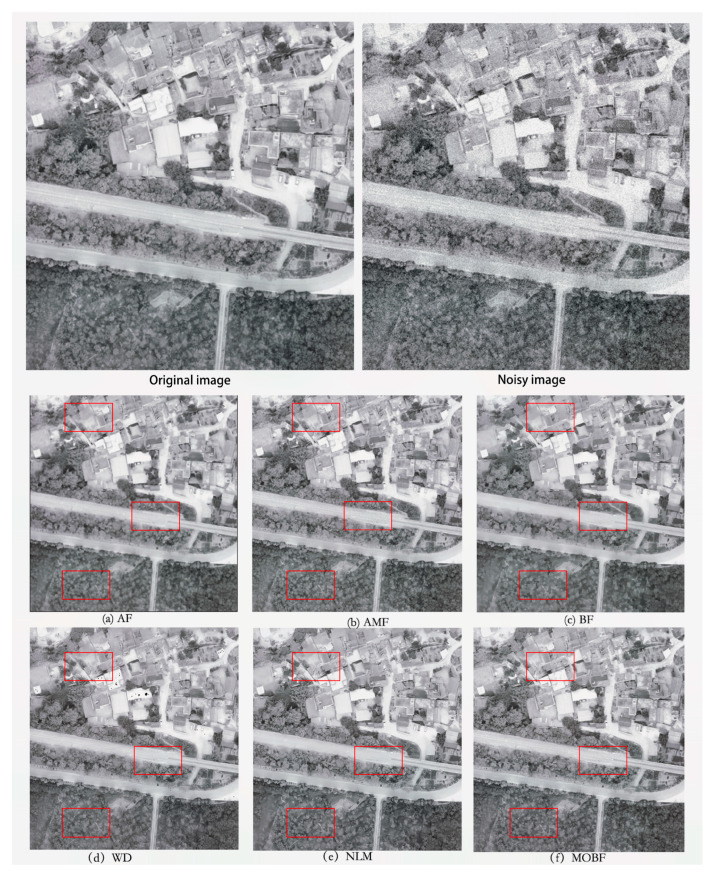
Comparison of UAV image denoising results.

Similarly, a simulation experiment was conducted on unmanned aerial vehicle (UAV) remote sensing images to assess the denoising effectiveness of different algorithms in terms of three metrics: the PSNR, the MSE, and the SSIM. The experiment’s results are presented in Table 2, and a comparative visualization of the denoising outcomes for UAV images is displayed in Figure 5.

**Figure 6:**
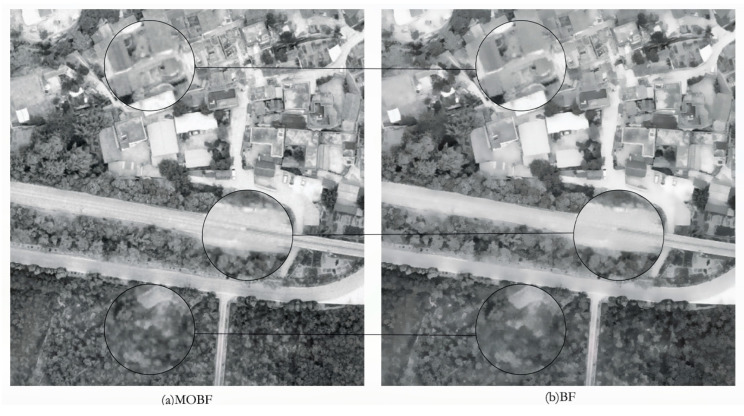
Comparison chart of BF and MOBF algorithms.

As can be seen in Figure 4, the AF, AMF, BF, WD, and NLM algorithms exhibited a considerably higher level of the smoothing effect within the rectangular box region after denoising, surpassing the performance of the MOBF algorithm. However, these algorithms sacrificed some texture details in the process. A further analysis and comparison of the BF and MOBF algorithms revealed that optimized bilateral filtering excels at preserving the edge details of building rooftops, roads, and vegetation areas. A comparison of the BF and MOBF algorithms is shown in Figure 6.

**Figure 7:**
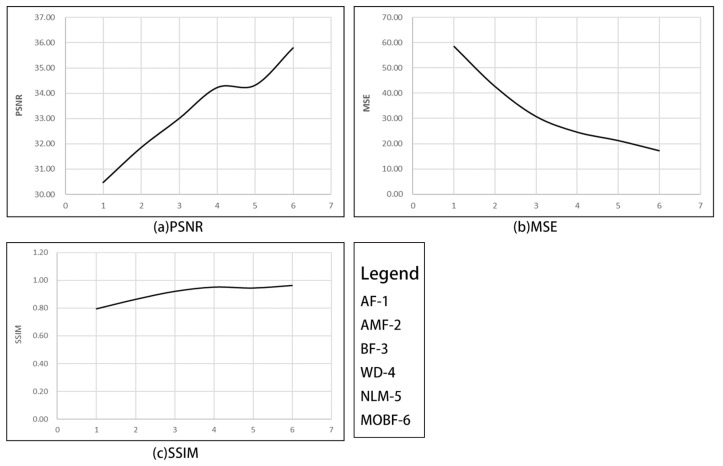
The average value of each algorithm evaluation index—*UAV image*.

As can be observed from the experimental results summarized in Table 2, the proposed MOBF algorithm outperformed the traditional BF, WD, and NLM algorithms for all three evaluation metrics, thus demonstrating that the remote sensing images processed using the MOBF algorithm exhibit an improved denoising performance and enhanced edge preservation. The algorithm performance is shown in Figure 7.

In the power spectral density (PSD) plot of an image, the low-frequency region corresponds to low-frequency information, the overall brightness, and the color; the mid-frequency region represents texture information, details, textures, and other intricate patterns within the image; and the high-frequency region reveals edge information, including the edges, contours, and other distinct features within the image.

**Figure 8:**
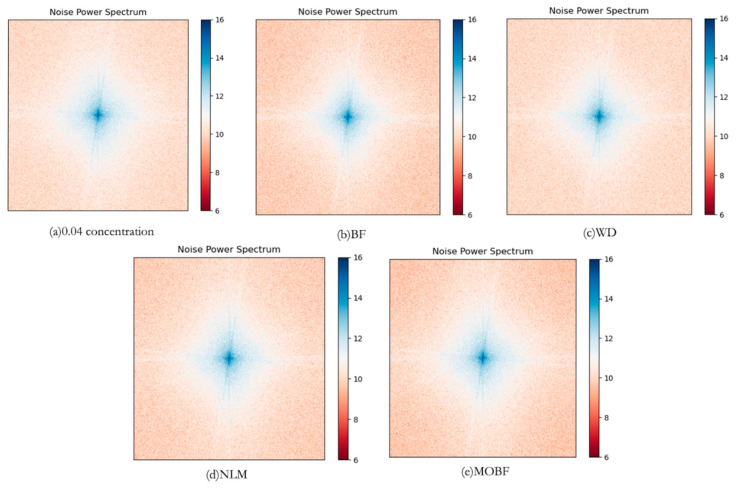
Noise power density diagrams.

In this study, a comparison of noise power spectral density was performed between the proposed algorithm and three filtering algorithms (BF, WD, and NLM). The power spectral density plots of the images after applying different processing methods are shown in Figure 8. The analysis of the power spectral density revealed that the information content in the low-, mid-, and high-frequency domains of the remote sensing images processed using the MOBF method was highly similar, thus indicating that the image modification did not result in a significant degradation of the texture, detail, or edge information. In the comparison of the MOBF algorithm with the BF algorithm, the higher energy of the MOBF algorithm in the mid-frequency region indicated better texture information preservation. In the comparison of the MOBF algorithm with the WD algorithm, the high-frequency region of the WD algorithm exhibited higher energy levels, exceeding even those of the original image, indicating that some noise remained uneliminated. The MOBF algorithm exhibited a higher energy level in the edge region compared to the NLM algorithm, thus demonstrating its superior edge preservation capability.

In the noise power spectral density plot, a high energy in the high-frequency region indicates the existence of high-frequency noise in the image, a high energy in the mid-frequency region indicates a considerable amount of detail and a lower level of image smoothness, and a higher energy in the low-frequency region is indicative of increased brightness and contrast in the image, leading to an overall brighter appearance. As can be observed from Figure 8, the denoising effectiveness was in the following order: MOBF algorithm > BF algorithm > NLM algorithm > WD algorithm.

## 6. Conclusions

In this paper, the MOBF algorithm was proposed to improve upon traditional bilateral filtering in terms of denoising effectiveness and edge texture preservation by utilizing edge detection operators and the DE evolutionary algorithm for multiple optimizations. The optimizations are made at the spatial domain filter kernel, spatial domain Gaussian kernel, and pixel range domain kernel. The proposed MOBF algorithm exhibited satisfactory performance, even in the absence of input parameters for the bilateral filtering algorithm.

The experiments detailed in this paper focus on varying levels of Gaussian noise intensity. The denoising experimental results for remote sensing images demonstrated the superior performance of the MOBF algorithm. Evaluation metrics such as the PSNR, the MSE, and the SSIM revealed the superiority of the MOBF algorithm over traditional approaches. Based on the outcomes of the experiments, the algorithm introduced in this study demonstrates enhancements in comparison to the optimal outcomes achieved by the comparative algorithms. Notably, there was a noteworthy 2.79% enhancement in the PSNR, a substantial 6.25% decrease in the MSE, and a discernible 1.05% improvement in the SSIM. The images processed using the MOBF algorithm exhibited remarkable denoising and edge preservation effects, resulting in enhanced image clarity.

These results validate the feasibility and effectiveness of the MOBF algorithm for denoising remote sensing images. Future research can further explore the algorithm’s applicability in other remote sensing scenarios and focus on enhancing its computational efficiency and practicality to drive advancements in the field of remote sensing image processing.

## Figures and Tables

**Table 1:** Evaluation index of GF-1 image denoising results of different algorithms.

Algorithm	PSNR/MES/SSIM			
	1%	2%	3%	4%
AF	33.147/31.502/0.889	32.739/34.605/0.872	32.395/37.456/0.856	32.119/39.917/0.844
AMF	35.271/19.321/0.938	34.581/22.644/0.922	34.026/25.729/0.906	33.627/28.202/0.895
BF	34.067/24.572/0.905	33.819/25.977/0.897	33.600/27.219/0.890	33.435/28.215/0.886
WD	35.196/16.654/0.935	35.084/20.169/0.934	35.078/20.197/0.935	34.907/21.066/0.935
NLM	34.468/21.634/0.920	34.014/23.895/0.909	33.621/26.072/0.900	33.321/27.723/0.893
MOBF	38.937/8.300/0.976	38.048/10.190/0.965	37.362/11.937/0.957	36.876/13.348/0.951

**Table 2:** Evaluation index of UAV image denoising results of different algorithms.

Algorithm	PSNR/MSE/SSIM			
	1%	2%	3%	4%
AF	30.940/52.350/0.827	30.560/57.110/0.803	30.310/60.520/0.780	30.100/63.520/0.768
AMF	32.440/37.040/0.889	31.950/41.470/0.868	31.640/44.590/0.853	31.400/47.060/0.839
BF	33.320/28.650/0.921	33.050/30.090/0.918	32.880/32.210/0.918	32.760/31.990/0.920
WD	34.390/23.630/0.949	34.340/23.890/0.950	34.090/25.320/0.950	34.080/25.370/0.949
NLM	34.780/20.160/0.949	34.040/22.660/0.939	34.120/21.760/0.941	34.330/20.390/0.944
MOBF	36.510/14.530/0.965	35.840/16.920/0.961	35.520/18.260/0.959	35.290/19.190/0.959

## Data Availability

All the data generated and analyzed during this study are included in this article.
